# Co-transfer of IncFII/IncFIB and IncFII plasmids mediated by *IS26* facilitates the transmission of *mcr-8.1* and *tmexCD1-toprJ1*

**DOI:** 10.1186/s12941-024-00676-5

**Published:** 2024-02-13

**Authors:** Qian Wang, Meng Zhang, Yue Liu, Jinmei Li, Ran Chen, Yueling Wang, Yan Jin, Yuanyuan Bai, Zhen Song, Xinglun Lu, Changyin Wang, Yingying Hao

**Affiliations:** 1 Department of Clinical Laboratory, Shandong Provincial Hospital Affiliated to Shandong First Medical University, No.9677 Jing-Shi Road, Jinan, 250021 Shandong People’s Republic of China; 2 Department of Clinical Laboratory, Shandong Provincial Hospital, Cheeloo College of Medicine, Shandong University, Jinan, 250021 Shandong China; 3 Department of Clinical Laboratory, Liaocheng Second People’s Hospital, Liaocheng, 252600 Shandong China; 4 Department of Clinical Laboratory, Jinan Seventh People’s Hospital, Jinan, 250021 Shandong China

**Keywords:** *Klebsiella pneumoniae*, *tmexCD1-toprJ1*, *Mcr-8.1*, *aac(3’)-IV*, Colistin, Tigecycline

## Abstract

**Purpose:**

This study aimed to characterise the whole-genome structure of two clinical *Klebsiella pneumoniae* strains co-harbouring *mcr-8.1* and *tmexCD1-toprJ1*, both resistant to colistin and tigecycline.

**Methods:**

*K. pneumoniae* strains TGC-02 (ST656) and TGC-05 (ST273) were isolated from urine samples of different patients hospitalised at separate times in 2021. Characterisation involved antimicrobial susceptibility testing (AST), conjugation assays, whole-genome sequencing (WGS), and bioinformatics analysis. Comparative genomic analysis was conducted on mcr-8.1-carrying and *tmexCD1-toprJ1*-carrying plasmids.

**Results:**

Both *K. pneumoniae* isolates displayed a multidrug-resistant phenotype, exhibiting resistance or reduced susceptibility to ampicillin, ampicillin/sulbactam, cefazolin, aztreonam, amikacin, gentamicin, tobramycin, ciprofloxacin, levofloxacin, nitrofurantoin, trimethoprim/sulfamethoxazole, apramycin, tigecycline and colistin. WGS analysis revealed that clinical strain TGC-02 carried the *TmexCD1-toprJ1* gene on a 200-Kb IncFII/IncFIB-type plasmid, while *mcr-8* was situated on a 146-Kb IncFII-type plasmid. In clinical strain TGC-05, *TmexCD1-toprJ1* was found on a 300-Kb IncFIB/IncHI1B/IncR-type plasmid, and *mcr-8* was identified on a 137-Kb IncFII/IncFIA-type plasmid. Conjugation experiments assessed the transferability of these plasmids. While transconjugants were not obtained for TGC-05 despite multiple screening with tigecycline or colistin, pTGC-02-tmex and pTGC-02-mcr8 from clinical *K. pneumoniae* TGC-02 demonstrated self-transferability through conjugation. Notably, the rearrangement of pTGC-02-tmex and pTGC-02-mcr8 via *IS26*-based homologous recombination was observed. Moreover, the conjugative and fusion plasmids of the transconjugant co-harboured the *tmexCD1-toprJ1* gene cluster and *mcr-8.1*, potentially resulting from *IS26*-based homologous recombination.

**Conclusion:**

The emergence of colistin- and tigecycline-resistant *K. pneumoniae* strains is concerning, and effective surveillance measures should be implemented to prevent further dissemination.

**Supplementary Information:**

The online version contains supplementary material available at 10.1186/s12941-024-00676-5.

## Introduction

*Klebsiella pneumoniae*, a member of the *Enterobacteriaceae* family, is a significant clinical species commonly associated with nosocomial infections such as pneumonia, bloodstream infection, urinary tract infection, and soft tissue infection [1]. It poses an emerging challenge for clinical settings worldwide due to the extensive use of antibiotics, leading to the emergence and rapid dissemination of multidrug-resistant *K. pneumoniae*, especially those resistant to last-line antibiotics such as carbapenems, colistin, and tigecycline [2].

Tigecycline, a semisynthetic glycylcycline derivative of tetracycline [3], is frequently employed to treat complex infections caused by multidrug-resistant Gram-positive and Gram-negative bacteria [4]. Tigecycline resistance in *K.* *pneumoniae* is driven by chromosomal mutations, including overexpression of efflux pumps or ribosomal mutations [5]. Additionally, a plasmid-borne resistance-nodulation division-type (RND)-type multidrug efflux pump gene cluster*, tmexCD1-toprJ1*, which confers resistance to tigecycline, quinolones, cephalosporins, and aminoglycosides, was initially identified in *K. pneumoniae* of animal origin [6] and later in clinical isolates [7]. Notably, *tmexCD1-toprJ1* has been detected in food-producing animals [6], human clinical isolates [8] and the environment [9, 10], indicating the widespread dissemination of this resistance.

Colistin is regarded as a last-line antibiotic, used either alone or in combination with other drugs to combat severe infections caused by carbapenem-resistant pathogens [11]. The extensive use of colistin in veterinary and human medicine has given rise to colistin resistance [12]. Plasmid-mediated colistin resistance (mcr) genes have extended colistin resistance horizontally among different species. The mobile colistin gene *mcr-8*, found on an IncFII-type conjugative plasmid in *K. pneumoniae* [13], has given rise to five identified variants (*mcr-8.1* to *mcr-8.5*) [14].

This *tmexCD1-toprJ1* gene cluster can be horizontally transferred along with the colistin resistance gene *mcr-8* and is primarily associated with *K. pneumoniae* [6]. In this study, we aimed to characterise the whole-genome structure of two clinical *Klebsiella pneumoniae* strains resistant to both colistin and tigecycline, co-harbouring *mcr-8.1* and *tmexCD1-toprJ1*, underscoring the convergence and co-transmission risk of these resistance genes.

## Materials and methods

### Patients and bacterial strains

We collected two *K. pneumoniae* isolates (TGC-02 and TGC-05) that exhibited resistance to both tigecycline and colistin. *K. pneumoniae* strain TGC-02 was isolated from a urine sample from a 79-year-old male patient diagnosed with prostatic hyperplasia. Initially, the patient sought treatment for urinary difficulties at a local hospital, which included the insertion of a urinary catheter. In October 2021, he was admitted to our hospital for further evaluation and management. However, surgery was ruled out due to underlying health issues. During his hospitalisation, he was diagnosed with a urinary tract infection and received treatment with cefuroxime sodium. Upon discharge, the patient regained the ability to urinate independently. *K. pneumoniae* strain TGC-05 was isolated from a urine sample obtained from a 28-year-old male patient with ureteral stones at the same hospital in August 2021. Initially, the patient was admitted to the urology department of our hospital in June 2021 due to left ureteral stones. He underwent lithotripsy, and a double-J stent was inserted, which was removed two months later. During a follow-up examination in August, the patient was diagnosed with a left epididymal cyst and left scrotal inflammation. Subsequently, the double-J stent was removed, and treatment with cefuroxime sodium was administered. The patient was discharged in good condition. The species of the isolate was determined using matrix-assisted laser desorption/ionisation time-of-flight mass spectrometry (MALDI-TOF/MS) (BioMérieux, France), and was confirmed by whole-genome sequencing (WGS).

### Antimicrobial susceptibility testing

The antibiotic susceptibility testing (AST) was conducted for a range of antibiotics, including aztreonam, cefepime, ceftriaxone, ceftazidime, ertapenem, imipenem, piperacillin/tazobactam, trimethoprim/sulfamethoxazole, ciprofloxacin, levofloxacin, gentamicin, amikacin, ampicillin, ampicillin-sulbactam, cefazolin, cefotetan, tobramycin, and nitrofurantoin. The VITEK-2 compact system (BioMérieux, France) was employed to perform these tests. The results were interpreted in accordance with the Clinical and Laboratory Standards Institute (CLSI) breakpoints (CLSI, 2022). Additionally, the minimum inhibitory concentrations (MICs) of tigecycline and colistin were determined using the broth microdilution, and the interpretations were made following the guidelines provided by the European Committee on Antimicrobial Susceptibility Testing (EUCAST, 2022).

### Conjugation assay

The transferability of tigecycline and colistin resistance genes was determined through a filter mating assay, using clinical strains resistant to tigecycline and colistin as donors and rifampin-resistant *Escherichia coli* C600 as the recipient. Transconjugants carrying different resistance plasmids were screened on Mueller–Hinton (MH) plates containing various antibiotics for conjugation assays. The antibiotics were combined as follows: tigecycline (4 µg/ml) and rifampin (2.5 mg/ml); colistin (2 µg/ml) and rifampin (2.5 mg/ml); and apramycin (30 µg/ml) and rifampin (2.5 mg/ml). Antibiotic susceptibility testing and PCR analysis were performed to confirm the transfer of plasmids carrying the *TmexCD1-ToprJ1, mcr-8.1* and/or *aac(3’)-IV* resistance genes. Specific PCR primers can be found in Additional file 1: Table S1.

### Whole-genome sequencing, assembly, and annotation

Total genomic DNA was extracted from the clinical isolates and transformants using a commercial genomic DNA kit (Qiagen, Hilden, Germany). Subsequently, genomic DNA sequencing was carried out employing the Illumina HiSeq platform (Novogene Co., Ltd., Beijing, China) and a PacBio RSII sequencer (Biozeron Biological Technology Co., Ltd., Shanghai, China). The paired-end short Illumina reads and long PacBio reads were subjected to hybrid assembling using Unicycler v0.5.0 [15] in normal mode.

The resulting genome sequences were annotated using Prokka [16] and Rapid Annotation Subsequencing Technology (RAST) (http://rast.nmpdr.org/), complemented by BLASTP/BLASTN searches(with a minimum identity of 90% and minimum coverage of 98%) across various specific databases, including PlasmidFinder [17], ResFinder [18], CARD [19], VirulenceFinder VFDB [20], ISFinder (https://isfinder.biotoul.fr/) and oriTfinder(https://bioinfo-mml.sjtu.edu.cn/oriTfinder/).

For comparative analsysism plasmid and genetic context comparisons were conducted using the BLAST Ring Image Generator (BRIG) [21] and Easyfig [22] tools, respectively.

### Pulsed-field gel electrophoresis

S1-pulsed-field gel electrophoresis (PFGE) was employed to validate both the size and number of plasmids present in the transconjugant and clinical strains. To achieve this, bacterial whole-cell DNA from the clinical isolates and their transconjugants was embedded in agarose plugs and subjected to digestion with S1 nuclease (Takara, Tokyo, Japan). As a reference marker, *Salmonella enterica* serovar Braenderup H9812, digested with *XbaI*, was utilised. The DNA fragments were separated using the CHEF-Mapper PFGE system (Bio-Rad) under the following conditions: 14 °C, 6 V/cm, and a 120° pulse angle for 16 h, with the initial and final pulses lasting 2.16 and 63.8 s, respectively. Subsequently, the PFGE results were analysed using InfoQuest software version 4.5 (Bio-Rad Laboratories, Hercules, CA, USA).

### Analysis of plasmids carrying mcr-8 and tmexCD1-toprJ1 genes within the NCBI database

Within the NCBI database (until September 2023), the complete sequences of *mcr-8* and *tmexCD1-toprJ1* were utilised for separate searches of complete sequences of plasmids bearing mcr-8 (coverage = 99%, identity = 100%) bearing and *tmexCD1-toprJ1* (coverage and identity > 96%). Typing of these plasmids were carried out using the PlasmidFinder [17]. Additionally, an analysis of *IS26* distribution among these plasmids was conducted through a local Blast search.

## Results

### Characterisation of *K. pneumoniae* isolates resistant to colistin and tigecycline

The two *K. pneumoniae* isolates exhibited identical antimicrobial resistance phenotypes and demonstrated resistance or non-susceptibility to most tested antibiotics, including ampicillin, ampicillin/sulbactam, cefazolin, aztreonam, amikacin, gentamicin, tobramycin, ciprofloxacin, levofloxacin, nitrofuran, trimethoprim/sulfamethoxazole, tigecycline, and colistin (Table 1). Based on WGS analysis, multiple drug resistance genes were identified in both strains, encompassing *mcr-8*, *tmexCD1-toprJ1, sul1*, *arr-3*, *catB3*, OXA-1, *AAC(6')-Ib-cr*, *bla*_CTX-M-55_, *QnrB20, AAC(3)-IV*, and others (Table 2). Therefore, these strains exhibited multidrug resistance.

**Table 1:** Antibiotic susceptibility testing of TGC-05, TGC-02 and the transconjugants

Strain	AMP	SAM	TZP	CZO	CTT	CRO	CAZ	FEP	ATM	ETP	IPM	AMK	GEN	TOB	CIP	LVX	SXT	TGC	COL
Clinical isolate																			
TGC-05	≥ 32R	≥ 32R	≤ 4S	≥ 64R	16S	≥ 64R	≥ 64R	2S	≥ 64R	≤ 0.5S	≤ 1S	≥ 64R	≥ 16R	≥ 16R	≥ 4R	≥ 8R	≥ 320R	8	32
TGC-02	≥ 32R	≥ 32R	8S	≥ 64R	≤ 4S	≥ 64R	16R	8SDD	≥ 64R	≤ 0.5S	≤ 1S	≥ 64R	≥ 16R	≥ 16R	≥ 4R	≥ 8R	≥ 320R	8	8
Recipient																			
EC600	8S	4S	4S	4S	4S	≤ 1S	≤ 1S	≤ 1S	≤ 1S	≤ 0.5S	≤ 1S	≤ 2S	≤ 1S	≤ 1S	< 0.25S	0.5S	≤ 20S	≤ 0.125	≤ 0.5
Transconjugant																			
JTGC-02-mcr8	8S	4S	≤ 4S	≤ 4S	≤ 4S	≤ 1S	≤ 1S	≤ 1S	≤ 1S	≤ 0.5S	≤ 1S	≤ 2S	≥ 16R	8I	1S	1S	≥ 320R	0.5	4
JTGC-02-Apr	16I	4S	≤ 4S	≤ 4S	≤ 4S	≤ 1S	≤ 1S	≤ 1S	≤ 1S	≤ 0.5S	≤ 1S	≤ 2S	≥ 16R	≥ 16R	0.5S	1S	≤ 20S	0.125	0.5
JTGC-02–1-Tmex	16I	4S	≤ 4S	≤ 4S	≤ 4S	≤ 1S	≤ 1S	≤ 1S	≤ 1S	≤ 0.5S	≤ 1S	≤ 2S	≥ 16R	≥ 16R	≥ 4R	2S	≥ 320R	0.5	2
JTGC-02–2-Tmex	16I	8S	≤ 4S	≤ 4S	≤ 4S	≤ 1S	≤ 1S	≤ 1S	≤ 1S	≤ 0.5S	≤ 1S	≤ 2S	≥ 16R	≥ 16R	≥ 4R	2S	≥ 320R	0.25	2

*MIC* Minimal inhibitory concentrations, *AMP* ampicillin, *SAM* ampicillin/sulbactam, *TZP* piperacillin/tazobactam, *CZO* cefazolin, *CTT* cefotetan, *CRO* cefatriaxone, *CAZ* ceftazidime, *FEP* cefepime, *ATM* aztreonam, *ETP* ertapenem, *IMP* imipenem, *AMK* amikacin, *CEN* gentamicin, *TOB* tobramycin, *CIP* ciprfloxacin, *LVX* levofloxacin, *SXT* trimethoprim/sulfamethoxazole, *TGC* tigecycline, *COL* colistin

**Table 2:** Characteristics of TGC-05, TGC-02 and the transconjugants genome components

Strains	MLST	Chromosome or plasmid	Size (bp)	Plasmid type	AMR genes
TGC-05	273	Chromosome	5,279,365	–	*CRP, emrR, oqxB, oqxA, acrD, baeR, mdtC, mdtB, H-NS, marA, SHV-11, msbA, ramA, acrA, acrB, FosA6, cpxA*
		pTGC-05-Tmex	309,063	IncFIB/IncHI1B/IncR	*aadA2, cmlA1, ANT(3'')-IIa, qacH,sul3, AAC(3)-IV, APH(4)-Ia, APH(6)-Id, APH(3'')-Ib, FosA3, TEM-141, CTX-M-55, APH(3')-Ia, mphE, msrE, armA, sul1, DHA-1, QnrB4, TmexCD1-toprJ1*
		pTGC-05-mcr8	137,567	IncFIA/IncFII	*MCR-8, floR, tet(A), mphA, QnrB20, sul1, aadA16, dfrA27, arr-3, AAC(6')-Ib-cr*
TGC-02	656	Chromosome	5,212,819	–	*CRP, emrR, oqxB, oqxA, acrD, baeR, mdtC, mdtB, H-NS, marA, SHV-187, msbA, ramA, acrA, acrB, FosA6, cpxA*
		pTGC-02-OXA	246,588	IncFIB/IncHI1B	*mphE, msrE, armA, sul1, arr-3, catB3, OXA-1, AAC(6')-Ib-cr, CTX-M-55*
		pTGC-02-Tmex	207,447	IncFIB/IncFII	*qacH, sul3, AAC(3)-IV, APH(4)-Ia, APH(6)-Id, APH(3'')-Ib, TmexCD1-toprJ1*
		pTGC-02-mcr8	146,089	IncFII	*MCR-8, sul2, APH(3'')-Ib, APH(6)-Id, APH(3')-Ia, AAC(3)-IId, AAC(6')-Ib-cr, arr-3, dfrA27, aadA16, sul1, QnrB20, mphA, tet(A), floR*
JTGC-02-2-Tmex	–	Chromosome	4,600,459	–	*gadX, gadW, mdtF, mdtE, CRP, acrF, acrE, acrS, bacA, tolC, emrB, emrA, emrR, mphB, acrD, ampC1, evgS, evgA, emrK, emrY, pmrF, yojI, baeR, baeS, mdtC, mdtB, mdtA, Ugd, marA, H-NS, mdtH, mdtG, msbA, mdfA, kdpE, emrE, acrA, acrB, ampH, mdtM, ampC, eptA, mdtN, mdtO, mdtP, cpxA*
		pJ-TGC-02–1	165,182	IncFII_1	*MCR-8, sul2, APH(3'')-Ib, APH(6)-Id, APH(3')-Ia, AAC(3)-IId, AAC(6')-Ib-cr, arr-3, dfrA27, aadA16, QnrB20, sul1, mphA, TmexCD1-toprJ1*
		pJ-TGC-02–2	188,350	IncFIB/IncFII	*qacH, sul3, AAC(3)-IV, APH(4)-Ia, tet(A), floR*

**Fig. 1:**
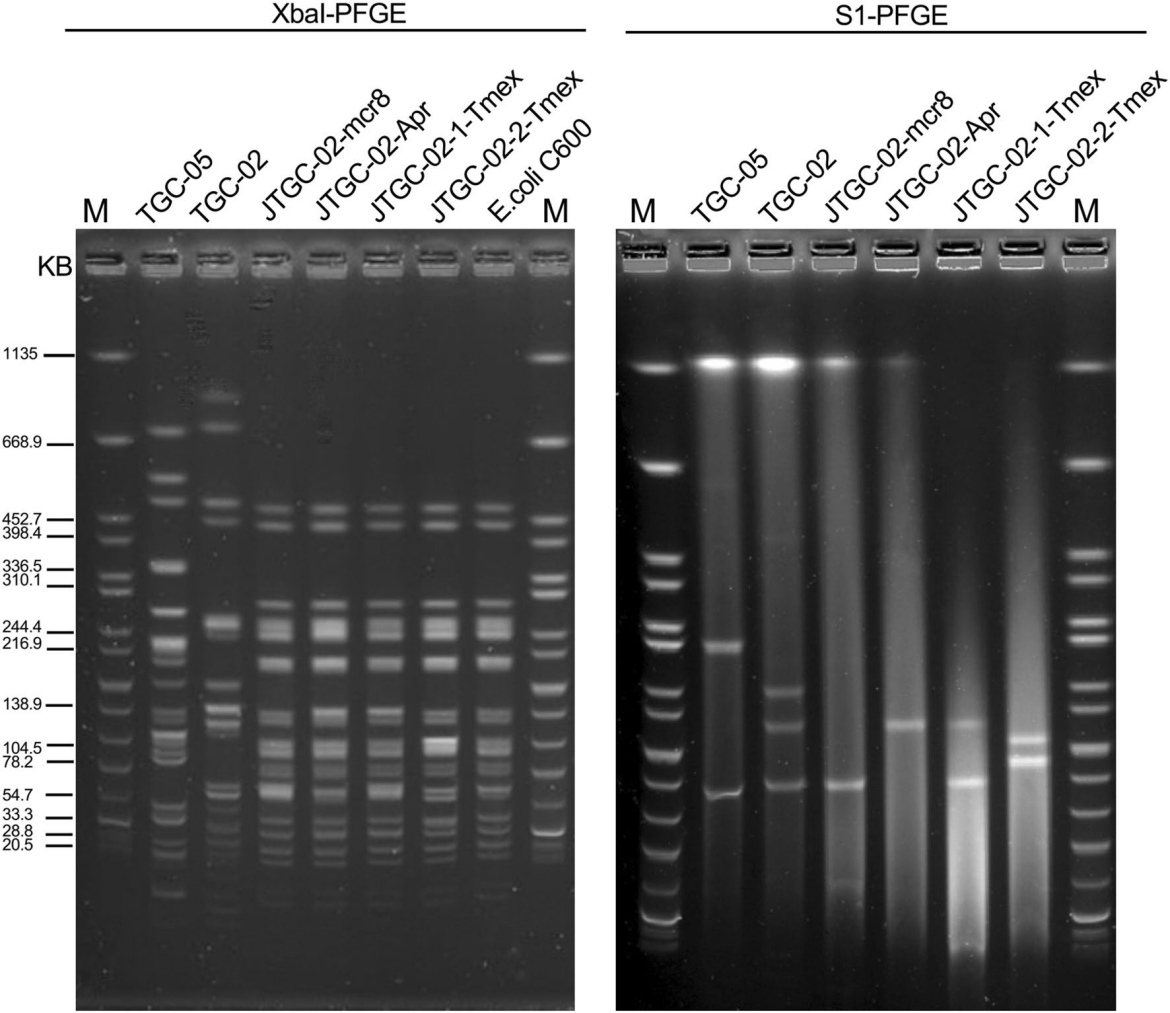
PFGE of wild-type *Klebsiella pneumoniae* TGC-02 and TGC-05, transconjugants, and *Escherichia coli C600* recipient strains

*K. pneumoniae* isolate TGC-02, assigned to ST656, harboured a chromosome (accession no. CP132219) and three plasmids, including pTGC-02-mcr8 (146.1 kb, accession no. CP132217) bearing *mcr-8*, pTGC-02-tmex (207.4 kb, accession no. CP132218) bearing *tmexCD1-toprJ1*. and pTGC-02-OXA (246.5 kb, accession no. CP132216). Conversely, *K. pneumoniae* isolate TGC-05, belonging to ST273, carried a chromosome (accession no. CP132220) and two plasmids, comprising pTGC-05-mcr8 (137.6 kb, accession no. CP132221) harbouring *mcr-8* and pTGC-05-tmex (309.1 kb, accession no. CP132222) bearing *tmexCD1-toprJ1* (Table 2). In line with these genomic features, S1-PFGE was employed to validate the size and number of plasmids in *K. pneumoniae* isolates TGC-02, TGC-05, and their transconjugants (Fig. 1).

### Analysis of Mcr-8 carrying plasmid

The plasmid pTGC-02-mcr8 comprised 146,089 base pairs (bp) plasmid and possessed an IncFII-type replicon. It consisted of two main regions: an approximately 70-kb backbone region, carrying a conjugal transfer region (traABCDEGHIKLMNQTUVWX) that promoted horizontal plasmid transfer among bacteria, and an approximately 70-kb drug resistance region encompassing antibiotic resistance genes, insertion sequences (ISs), and transposons. In the conjugation assay, pTGC-02-mcr8 demonstrated self-transferability. The resulting transconjugant JTGC-02-mcr8, displayed susceptibility to nearly all tested antibiotics, except for colistin, trimethoprim/sulfamethoxazole, gentamicin, and tobramycin (Table 1).

**Fig. 2:**
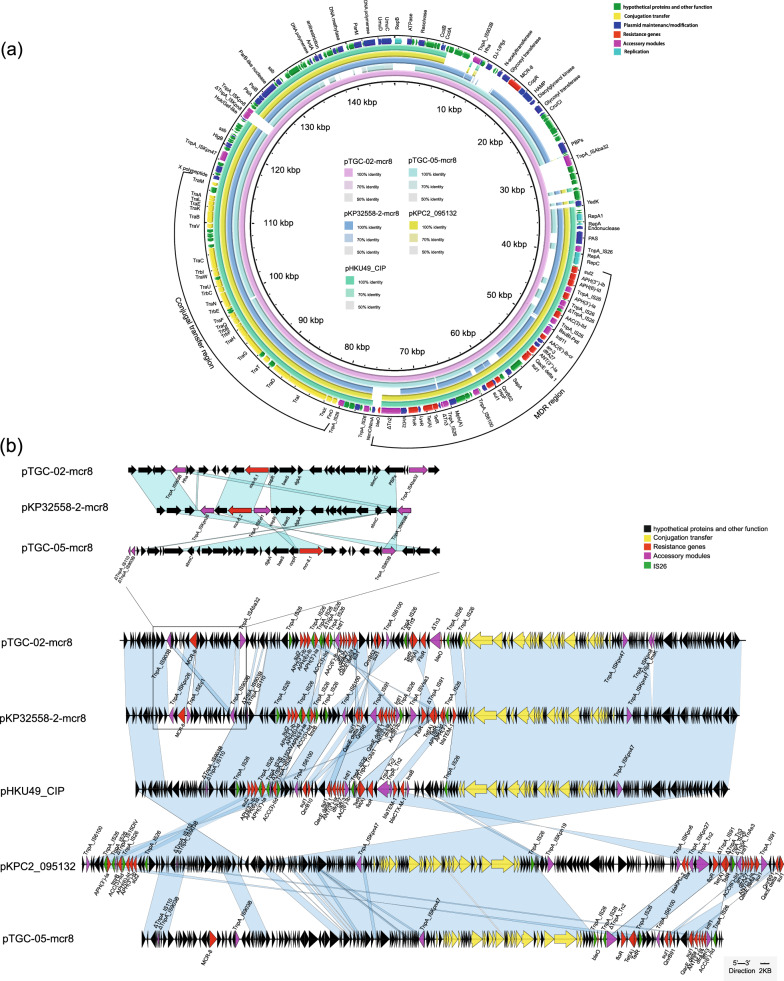
Genomic Characterisation of Plasmids Carrying *mcr-8.1*. The concentric circles, starting from the outermost to the innermost, depict the following information: the locations of predicted forward coding sequences (CDS), related plasmids, sequence positions in base pairs, GC skew curve, and GC contents. Different gene functions are color-coded as described in the legend. **a** Circular comparison between the *mcr-8.1*-positive plasmid pTGC-02-mcr8 and other similar plasmids. **b** Linear alignment of plasmids pKP32558-2-mcr8, pTGC-02-mcr8, and pTGC-05-mcr8, illustrates the genomic context surrounding the *mcr-8*. Additionally, the alignment of plasmids pKP32558-2-mcr8, pHKU49_CIP, and pKPC2_095132 with pTGC-02-mcr8 and pTGC-05-mcr8 from TGC-02 and TGC-05 reveals various functions denoted by arrows: black for proteins with other functions, yellow for conjugation transfer, red for resistance genes, green for IS26 elements, and rose for accessory modules

The BLAST query of the full pTGC-02-mcr8 sequence against the NCBI database indicated its similarity to pKP32558-2-mcr8(CP076032), pKPC2_095132(CP028389), and pHKU49_CIP(MN543570), with coverage ranging from 80 to 87% and identity exceeding 99.54%–99.86% (Fig. 2a). Further analysis revealed the presence of an entire *IS903B* insertion sequence, originating from *E. coli*, situated upstream of *mcr-8.1* in pTGC-02-mcr8, while an *ISAba32* transposon originally found next to the pdif site in the abkAB dif module, was identified downstream of *mcr-8.1* [23]. Additionally, the drug resistance region contained eight *IS26* genes, one *IS6100*, one *Intl1* and a *ΔTn3*. Beyond *mcr-8.1* gene, other antimicrobial resistance genes detected on the same plasmid include sulfonamide resistance genes *sul1* and *sul2*, aminoglycoside resistance genes *aac(3)-IId,aac(6’)-Ib-cr,aph(3’)-Ib,aph(6’)-Id* and *aadA16*, quinolone-resistant gene *qnrB20*, macrolide resistance gene *mphA,* trimethoprim resistance gene *dfrA27*, tetracycline resistance genes *tetR* and *tet(A)*, and rifampicin resistance ribosyltransferase gene *arr-3* (Fig. 2c).

The *mcr-8.1* gene in TGC-05 was located on an approximately 137.5-kb plasmid known as pTGC-05-mcr8, possessing a GC content of 51.2%. This plasmid belonged to the IncFII/IncFIA multiple replicon plasmid. While oriTfinder analysis revealed the presence of a complete type IV secretion system (T4SS) in pTGC-05-mcr8, several attempts at conjugation assays failed to yield transconjugants carrying pTGC-05-mcr8 (Fig. 2b). Although pTGC-05-mcr8 harbored almost identical T4SS with pTGC-02-mcr8, as assigned according to oriTfinder web tool, the cluster from FinO to TraF (104,246 bp to 87,266 bp) was with related low identity of 94% with that of pTGC-02-mcr8 (Additional file 3: Fig. S1). It was speculated that the mutations and rearrangement of lead to the loss of self-transferability capacity. The plasmid backbone of pTGC-05-mcr8 exhibited similarities to those of plasmids pKP32558-2-mcr8, pKPC2_095132 and pHKU49_CIP, with coverage ranging from 70 to 78% and identity exceeding 99.62%–99.75%. Structurally, the genetic environment surrounding *mcr-8.1* in pTGC-05-mcr8 consisted of *ΔIS110-ΔIS903B-orf-mcr-8.1-orf-IS903B*. In addition to *mcr-8.1*, pTGC-05-mcr8 also carried other resistance genes, including *tet(A)* (tigecycline efflux pump gene), *floR* (florfenicol resistance), *aac(6’)-Ib-cr* (aminoglycosides resistance), and *sul1*(sulfonamide resistance).

Comparative analysis revealed that both pTGC-02-mcr8 and pTGC-05-mcr8 shared an almost identical genetic structure with pKP32558-2-mcr8, which was previously reported in a lung transplant patient in China (Fig. 2b). Notably, pKP32558-2-mcr8 originated from ST656 *K. pneumonia*e [24] and shared a common ancestor with two mcr-8 carrying plasmids, pMCR8_020135(CP037964) and pMCR8_095845(CP031883). Our study suggested that *mcr-8*, carried on IncFII-type plasmids, shared a backbone structure similar to that of mcr-8 negative plasmids pKPC2_095132 and pHKU49_CIP, which also harboured various resistance genes. We compared the mcr-8 region on plasmid pTGC-02-mcr8, pTGC-05-mcr8 with the analogous region on plasmid pKP32558-2-mcr8 (Fig. 2b). The mcr-8 locus on pKP32558-2-mcr8 was characterized in previous research as comprising of *ISKpn26-orf-mcr8.2-ISEcl1-copR-baeS-dgkA*, with nearby *IS903B* [25]. Contrarily, the mcr8 region of plasmid pTGC-02-mcr8 appeared to lack *ISKpn26* and *ISEcl1*, while an *ISAba32* transposon was identified downstream of *mcr-8.1.* Additionally, the analogous region on plasmid in pTGC-05-mcr8 consisted of *ΔIS110-ΔIS903B-orf-mcr-8.1-orf-IS903B.*This observation implied that these plasmids may have evolved by acquiring mcr-8 under antibiotic pressure during transmission.

### Analysis of tmexCD1-toprJ1-carrying plasmid

**Fig. 3:**
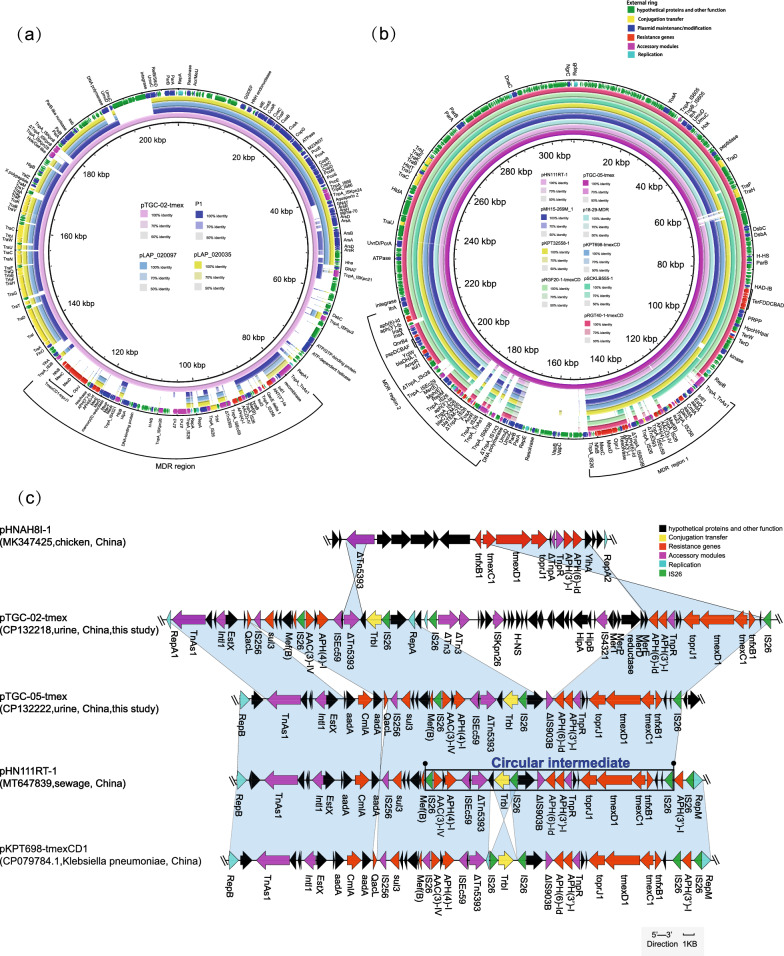
Genomic Analysis of Plasmids Carrying *tmexCD1-toprJ1*. Gene extents and orientations are indicated by arrows labeled with gene names, with tmexCD1-toprJ1 genes and resistant genes highlighted in red. Insertion sequences (ISs) are shown in purple, hypothetical protein genes in green, and other genes in blue. Horizontal lines represent the plasmid backbone, while black boxes denote the genetic structure of the tmexCD1-toprJ1-carrying region and its circular intermediate. **a** Circular comparison between the *tmexCD1-toprJ1*-bearing plasmid pTGC-02-tmex and other plasmids with resembling backbones in the NCBI database. The outermost red circle denotes the reference plasmid pTGC-02-tmex. Comparison between the genetic context of *tmexCD1-toprJ1* and those of closely related sequences, including pTGC-05-tmex, pLAP2_020097, pLAP2_020035, and P1. **b** Circular genetic mapping of *tmexCD1-toprJ1*-carrying plasmids, including pTGC-05-tmex, along with other similar plasmids, including pTGC-05-tmex, pKPT698-tmexCD (CP079784), pRGT40-1-tmexCD (CP075551), pSCKLB555-1(CP043933), pMH15-269M_1(AP023338), p18-29-MDR(MK262712), pHN111RT-1(MT647838), pRGF20-1-tmexCD (CP075455), and pKP32558-1(CP076031). **c** A linear comparative analysis was conducted to assess the genetic context of tmexCD1-toprJ1 in relation to closely related sequences, which included pHNAH8I-1 (accession: MK347425, a representative plasmid housing an RND efflux pump responsible for tigecycline resistance), *K. pneumoniae* pTGC-02-tmex (this study, accession CP132218), *K. pneumoniae* pTGC-05-tmex (this study, accession CP132221), pHNG11RT-1 (accession: MT637839), and pKPT698-tmexCD1 (accession: CP079784.1). Gene extents and orientations were represented by arrows labeled with gene names. The tmexCD1-toprJ1 genes and resistance-related genes were highlighted in red. ISs were indicated in purple, *IS26* elements in green, conjugation transfer genes in yellow, and other genes in black. The plasmid backbone was symbolized by horizontal lines, and the genetic structure of the region carrying *tmexCD1-toprJ1* and its circular intermediate was denoted by black boxes

Plasmid pTGC-02-tmex derived from strain TGC-02 was categorised as an IncFII/IncFIB-type plasmid. It was positive for *AAC(3)-IV* and *tmexCD1-toprJ1* and shared a similar backbone with previously published *tmexCD1-toprJ1* negative plasmids (namely, plasmids P1(OW969593), pLAP_020097(CP043350), and pLAP_020035(CP045991), exhibiting coverage ranging from 60 to 62% coverage and identity exceeding 99.79%–99.91% (Fig. 3a). Sequence analysis of pTGC-02-tmex revealed the presence of two metal resistance gene clusters, the *silE-cusS-cusR-cusC-cusF-cusB-cusA-copG* cluster, and the *copBCD* operon. A multidrug resistance region (MDR) contained many kinds of resistance genes such as *sul3, APH(4)-Ia, APH(6)-Id*, *APH(3’)-Ib*,and *tmexCD1-toprJ1.* In addition, multiple mobile genetic elements were identified, including four *IS26* copies, single *ISKpn24, ISKpn21,ISKqu3,TnAs1, Intl1*,,*IS256, ISEc59,ΔTn5393,ISKpn26,IS4321,ISKpn28,ISKpn8* and two partial *Tn3* structures (Fig. 3a). The gene context of *tmexCD1-toprJ1* on pTGC-02-tmex (*IS26*-*ΔTn3-ISKpn26-IS4321-[APH(6)-Id]-[APH(3’)-I]-tnpR-toprJ1-tmexD1-tmexC1-tnfxB1-IS26*) mirrored that on IncFIB/HI1B-type plasmid pHN111RT-1, with the exception of an approximately 20-kb segment [RepA-*IS26-ΔTn3-SKpn26-HipAB-IS4321-MerRTPDE*] inserted (Fig. 3c).

Plasmid pTGC-05-tmex, originating from strain TGC-05, harboured three replicon genes of the IncFIB/IncHI1B/IncR hybrid type and possessed a size of 309-kb. This plasmid contains two MDR regions, with the t*mexCD1-toprJ1* gene cluster situated within MDR region 1. Multiple mobile genetic elements, including three copies of *IS26*, a single *TnAs1*, I*S256, ISEc59, ΔIS903B*, and *Intl1*, were identified in MDR region 1 (Fig. 3b). Furthermore, MDR region 2 also harboured various genes conferring resistance to diverse antimicrobial agents, such as *FosA*, *bla*_*TEM-214*_*, bla*_*CTX-M-55*_*, APH(3’)-Ia, msr(E), mph(E), sul1, bla*_*DHA-1*_, *qnrB4*, and *APH(6)-Id* (Fig. 3b). The genetic structure encompassing the *tmexCD1-toprJ1* gene cluster on pTGC-05-tmex is *IS26-[aac(3)-IV]-[APH(4)-I]-ISEc59-ΔTn5393-TrbI-IS26-ΔIS903B-[APH(6)-Id]-[APH(3’)-I]-toprJ1-tmexD1-tmexC1-tnfxB1-IS26* and is 100% identical to the 20-kb circular intermediate of plasmid pHN111RT-1 (MT647839, *K. pneumoniae*, sewage, China) (Fig. 3c).

To assess the transferability of the *tmexCD1-toprJ1*-bearing plasmid pTGC-05-tmex from TGC-05, conjugation assays were employed using *Escherichia coli* J53, *Escherichia coli* C600, and *K. pneumoniae* NTUH-K2044 as recipients but were unsuccessful in obtaining transconjugants following several attempts. Likewise, electrotransformation and chemical transformation of plasmid pTGC-05-tmex into *E. coli DH5a* as the recipient yielded no positive results. In silico analysis of the T4SS using the oriTfinder web tool confirmed that the oriT sequence of pTGC-05-tmex was partially deleted, which might explain the non-transferability of this plasmid.

Fortunately, the tigecycline-resistant transconjugants TGC-02 were obtained by screening plates containing apramycin or tetracycline. Based on the S1-PFGE profile, the transconjugant JTGC-02-Apr, selected from the apramycin-supplemented plate, contained a single plasmid of the same size as the *tmexCD1-toprJ1* harbouring plasmid in the clinical strain TGC-02. Interestingly, the transconjugants obtained from the tigecycline-supplemented plate exhibited two distinct states: one transconjugant, JTGC-02-1-Tmex, carried two plasmids with sizes of approximately 200 kb and 140 kb, resembling the *tmexCD1-toprJ1*-harbouring plasmid pTGC-02-tmex and the mcr-8.1-harbouring plasmid pTGC-02-mcr8. The other transconjugant, JTGC-02-2-Tmex, contained two novel plasmids with sizes of approximately 180 kb and 160 kb (Fig. 1). Plasmid recombination was suspected during the formation of the transconjugant JTGC-02-2-Tmex.

To elucidate the mechanism of transmission and rearrangement of resistance plasmids, WGS and annotation of JTGC-02-2-Tmex were conducted.

### IS26-mediated arrangement of plasmids harbouring tmexCD1-toprJ1 and mcr-8

**Fig. 4:**
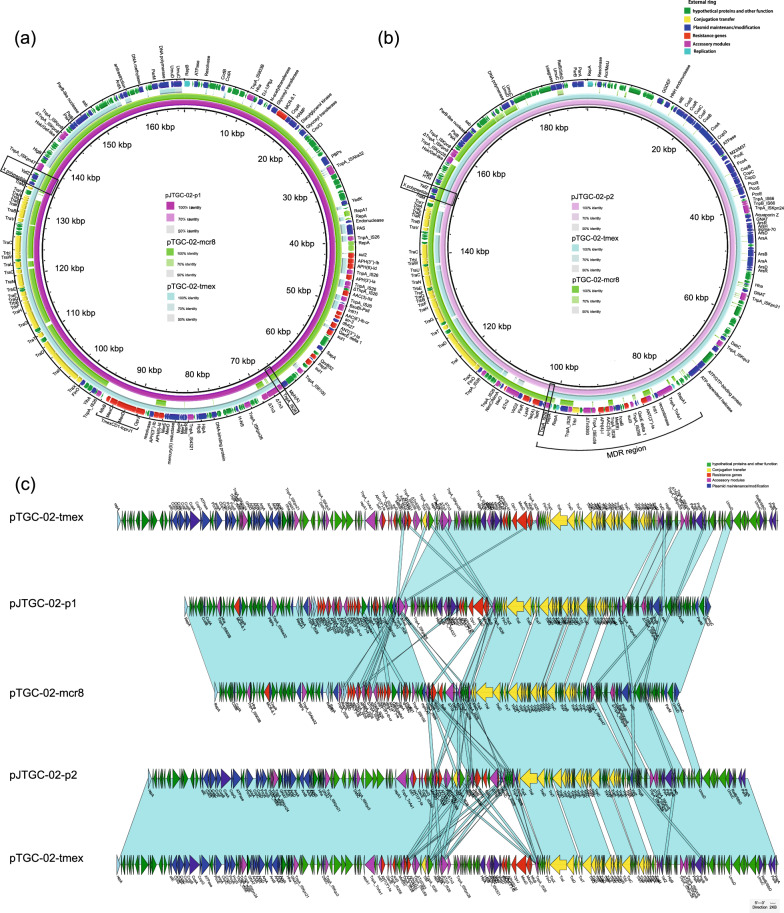
Genomic Analysis of Plasmids pJTGC-02-p1 and pJTGC-02-p2 in Transconjugant Strain JTGC-02–2-Tmex. **a** In circular comparison, we examine plasmid pJTGC-02-p1, which carries both mcr-8.1 and *tmexCD1-toprJ1*, along with plasmids pTGC-02-mcr8 and pTGC-02-tmex from clinical strain TGC-02. The plasmid fusion site is denoted by the black box. **b** Circular comparison involves the plasmid pJTGC-02-p2, co-harboring *tet(A)* and *AAC(3)-IV*, as well as plasmids pTGC-02-mcr8 and pTGC-02-tmex from clinical strain TGC-02. The plasmid fusion site is indicated by the black box. **c** A linear comparative analysis to illustrate the formation of transconjugants

The complete genome of the JTGC-02-2-Tmex strain revealed the presence of two plasmids, designated as pJTGC-02-p1 (accession no. CP132224) and pJTGC-02-p2 (accession no. CP132225) (Fig. 4). The first plasmid, pJTGC-02-p1, with a size of 165,182 bp, co-harboured *mcr-8.1* and *tmexCD1-toprJ1* genes. It appears to be a cointegration plasmid that likely originated from the progenitor IncFII-type plasmid pTGC-02-mcr8 and the IncFII/IncFIB-type plasmid pTGC-02-tmex (Fig. 4a and c). The second plasmid, pJTGC-02-p2, carried *tet(A)* and *AAC(3)-IV* and had a size of 188,350-bp. The plasmid backbone of pJTGC-02-p2 resembled that of the wild-type plasmid, pTGC-02-tmex, while the conjugal transfer and partially MDR regions matched those of plasmid pTGC-02-mcr8 (Fig. 4b and c).

**Fig. 5:**
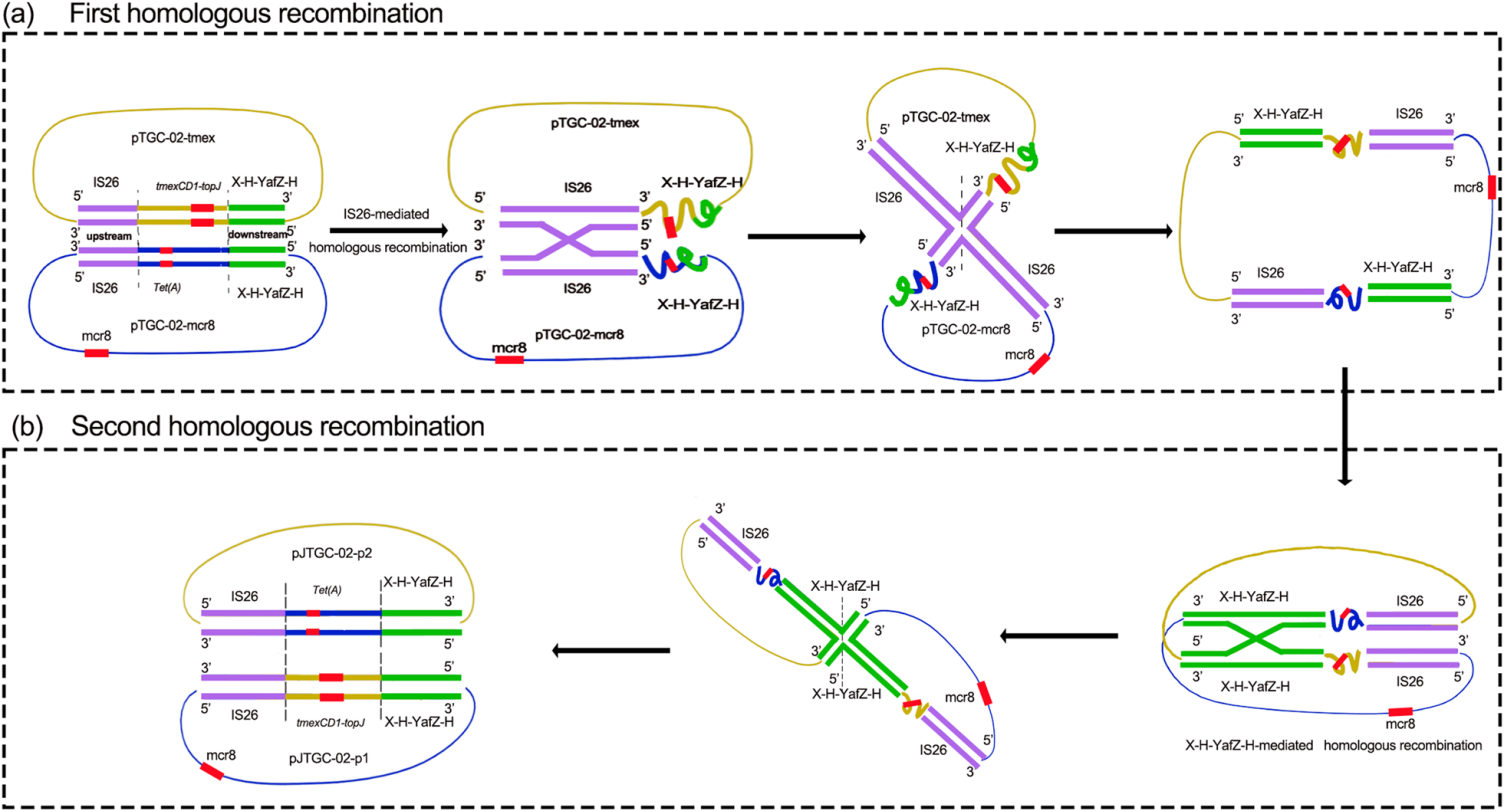
Hypothesized Mechanism of Plasmid Formation through Two Rounds of Homologous Recombination. The formation of pJTGC-02-p1 and pJTGC-02-p2 plasmids occurred through two rounds of homologous recombination. **a** The first-round homologous recombination, driven by *IS26,* resulted in the fusion of pTGC-02-tmex and pTGC-02-mcr8, creating a giant fusion plasmid. **b** The second-round homologous recombination, involving an identical sequence downstream of *tmexCD1-toprJ1* and *Tet(A)* regions, led to the dissociation of the fusion plasmid into the two novel plasmids, pJTGC-02-p1 and pJTGC-02-p2. This process replaced the *Tet(A)* region with the *tmexCD1-toprJ1* region, giving rise to pJTGC-02-p1, which co-harbors *mcr-8* and *tmexCD1-toprJ1.* pTGC-02-tmex is represented in yellow, pTGC-02-mcr8 in blue, *IS26* in purple double strands, X-H-YafZ-H in green double strands, and antibiotic resistance genes in red rectangles

It is hypothesized that the formation of these two novel plasmids, pJTGC-02-p1 and pJTGC-02-p2, occurred through two rounds of homologous recombination (Fig. 5). The first round of homologous recombination, based on *IS26* located upstream of the *tmexCD1-toprJ1* region (101,949–169,465 bp, including conjugation transfer cluster and partial MDR) on the pTGC-02-tmex plasmid and upstream of the *Tet(A)* region (66,965–11,5375 bp, including conjugation transfer cluster, partial MDR and a segment of backbone region) on the pTGC-02-mcr8 plasmid, resulted in the formation of a giant fusion plasmid. The second round of homologous recombination, based on an about 2 KB identical sequence composed by *X polypeptide-hypothetical protein-YafZ-hypothetical protein* (X-H-YafZ-H) located downstream of the *tmexCD1-toprJ1* gene cluster on the pTGC-02-tmex plasmid and downstream of the *Tet(A)* region on the pTGC-02-mcr8 plasmid, caused the dissociation of the fusion plasmid into two novel plasmids pJTGC-02-p1 and pJTGC-02-p2. In summary, the two rounds of homologous recombination resulted in the replacement of the *Tet(A)* region on the pTGC-02-mcr8 plasmid with the *tmexCD1-toprJ1* region on the pTGC-02-tmex plasmid, leading to the formation of pJTGC-02-p1 co-harboring *mcr-8* and *tmexCD1-toprJ1*.

### IS26 was commonly detected on plasmids carrying mcr-8 and tmexCD1-toprJ1

**Fig. 6:**
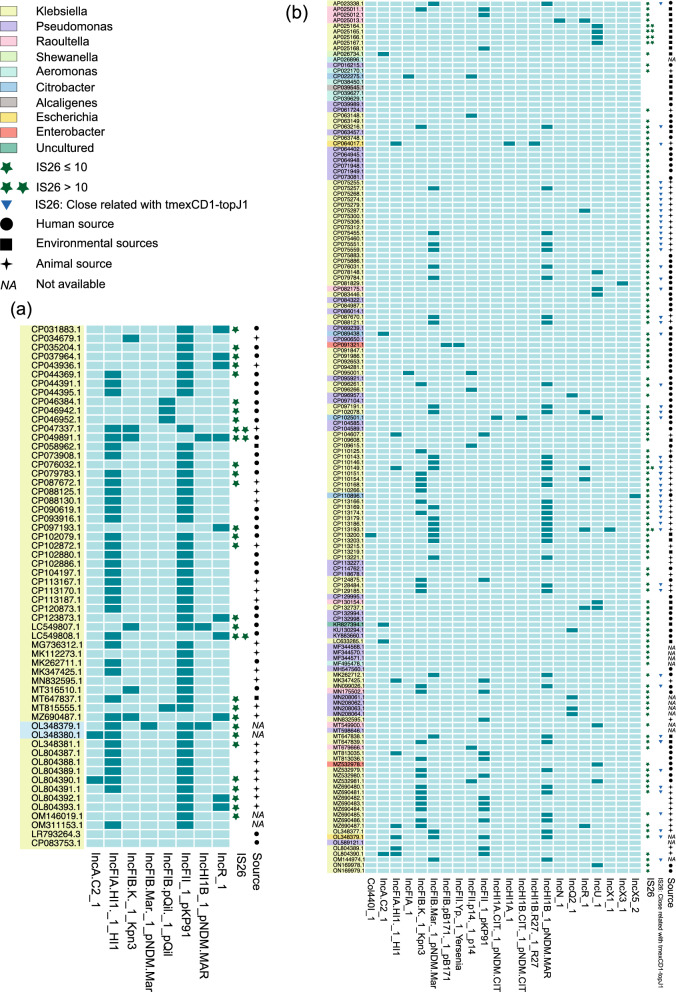
Matrixes of Plasmids Carrying *mcr-8* and *tmexCD1-toprJ1*. The presence and absence of replicons are denoted by dark green and light green boxes, respectively

A total of 58 complete sequences of mcr-8-bearing plasmids were obtained, with 56 sequences of them originating from *Klebsiella* species (Fig. 6a). Among the mcr-8-bearing plasmids, 28 of them were from human origin. According to plasmid typing results, 34 of these plasmids belonged to the IncFII/IncFIA type, and 6 plasmids carried at least one copy of IS26. Regarding *tmexCD1-toprJ1*-bearing plasmids, a total of 156 complete sequences were acquired, with 94 originating from *Klebsiella* species and 37 from *Pseudomonas* species (Fig. 6b). Among the *tmexCD1-toprJ1*-8-bearing plasmids, 62 of them were from human origin. Plasmid typing results indicated that 42 plasmids belonged to the IncFIB/IncHI1B type, and 127 plasmids harboured at least one copy of *IS26*. Among the 127 plasmids, 53 contain at least one copy of *IS26* within 2 KB proximity to *tmexCD1-toprJ1*, potentially facilitating the spread of *tmexCD1-toprJ1*. Additionally, among the 156 *tmexCD1-toprJ1*-bearing plasmids, 9 of them co-carried *tmexCD1-toprJ1* and *mcr* gene (Additional file 2: Table S2).

## Discussion

Colistin and Tigecycline are often considered the last line of defense against life-threatening infections caused by multidrug-resistant gram-negative pathogens. A recent nationwide surveillance study in China revealed a low occurrence of *tmexCD-toprJ* positive clinical Klebsiella spp. (7/2795, 0.25%) [26]. However, there is growing concern about the emergence of transmissible plasmids carrying *tmexCD1-toprJ1*, especially when they facilitate the co-transfer of *tmexCD1-toprJ1* and *mcr* genes through the same or different plasmids [7, 27–31]. In our study, we present findings on two clinical strains that carry both *TmexCD1-toprJ1* and *mcr-8* genes, aiming to uncover the mechanisms behind the accumulation of resistance. The most significant discovery in our research is the observed co-transfer of *TmexCD1-toprJ1* and *mcr-8* via the formation of novel hybrid plasmids mediated by *IS26*.

Previous research has suggested that *tmexCD1-toprJ1*-mediated tigecycline resistance primarily originates in chickens, as evidenced by the significant disparity in the prevalence of *tmexCD1-toprJ1*-positive strains between animal and human sources (52.4% vs. 2.5%) [26]. Alarmingly, approximately one-third of *tmexCD-toprJ* positive Klebsiella spp. were found to carry colistin resistance genes [26]. Our study, based on public databases, indicates that around 32.7% and 39.7% of *tmexCD1-toprJ1*-positive plasmids are of animal origin and human origin, respectively, while approximately 41.4% and 48.3% of *mcr-8*-positive plasmids are of animal and human origins. These seemingly contradictory findings may serve as evidence that *tmexCD1-toprJ1*-positive and *mcr-8*-positive plasmids are rapidly spreading from animals to humans. The prevailing plasmid types associated with *tmexCD1-toprJ1* and *mcr-8* are IncFIB/IncHI1B and IncFII/IncFIA, respectively. Among the 156 *tmexCD1-toprJ1*-bearing plasmids, 9 of them co-carried both *tmexCD1-toprJ1* and mcr genes. The co-occurrence of *tmexCD-toprJ* with mcr genes on the same plasmid poses a significant challenge for clinical management and potentially accelerates their dispersion.

*IS26* is well-studied and known to create clusters of antibiotic resistance genes interspersed with directly oriented genes, a phenomenon observed in multi-resistant pathogens. It is frequently reported to generate a translocatable unit (TU) element, facilitating the transfer of a single *IS26* copy along with an adjacent DNA segment to a new location [28]. This process can result in the deletion or inversion of DNA segments through a replicative route, as observed in previous studies [28]. Wan et al. suggested that *tmexCD1-toprJ1* may integrate into pHN111RT-1 through *IS26*-mediated cointegration with a circular intermediate, highlighting the potential for *tmexCD1-toprJ1* transmission among different plasmids and strains [9]. We hypothesize that the approximately 30-kb *tmexCD1-toprJ1*-associated fragment could have integrated into the IncFII plasmid pTGC-02-tmex via *IS26*-based homologous recombination during transmission. Further research is needed to explore the formation of circular intermediates within the *tmexCD1-toprJ1*-associated fragment.

*IS26* is capable of forming cointegrates through two mechanisms: the copy-in route, where cointegrates form between DNA molecules containing *IS26*, and the targeted conservative route, where recombination targets one or both ends of the IS elements [32]. Wang et al. reported that plasmids carrying mcr-1 could be co-transferred with plasmids containing *bla*_NDM-1_ or *tmexCD1-toprJ1* through plasmid hybridization [31]. Based on whole-genome sequencing (WGS) and S1-PFGE analyses, it is suggested that targeted conservative recombination of plasmids is likely mediated by *IS26* elements located on plasmids carrying *mcr-1*, *bla*_NDM-1_, or *tmexCD1-toprJ1* [31]. Cointegration via the conservative route occurs at a frequency over 50 times higher than that of copy-in cointegrate formation [32]. According to our research, *IS26* is common to both *mcr-8* and *tmexCD1-toprJ1*-bearing plasmids, increasing the likelihood of cointegration events and co-transferability. Consequently, further *IS26*-mediated mobilization of *tmexCD1-toprJ1* and *mcr-8* among different plasmid types remains possible. These events may contribute to the rapid and widespread accumulation of tigecycline and colistin resistance in Enterobacteriaceae, especially in the face of antibiotic selective pressures in the future.

## Conclusion

The simultaneous emergence of mobilized colistin and tigecycline resistance genes in clinical isolates is a concerning evolutionary trend. *IS26* is widely distributed on *mcr-8* and *tmexCD1-toprJ1*-bearing plasmids, which may accelerate the formation of colistin- and tigecycline-resistant strains and the creation of new hybrid plasmids. Therefore, it is imperative to enhance monitoring efforts to prevent the further spread of colistin- and tigecycline-resistant *Klebsiella pneumoniae* in healthcare settings.

### Supplementary Information

**Additional file 1: Table S1.** List of primers used in this study.

**Additional file 2: Table S2.** Plasmids Carrying mcr-8 and tmexCD-toprJ with Related Information.

**Additional file 3: Figure S1.** Comparative Analysis of conjugation transfer region. Alignment of the conjugation transfer region in pTGC-02-mcr8 and pTGC-05-mcr8.

## Data Availability

Data will be made available on request.

## Abbreviations

AST: Antimicrobial susceptibility testing
WGS: Whole-genome sequencing
RND: Resistance-nodulation division
MICs: Minimum inhibitory concentrations
CLSI: Clinical and Laboratory Standard Institute
MH: Mueller–Hinton
EUCAST: European Committee on Antimicrobial Susceptibility Testing
MDR: Multidrug resistance region
T4SS: Type IV secretion system
CDS: Coding sequences
ISs: Insertion sequences
PFGE: Pulsed-field gel electrophoresis
TU: Translocatable unit
