# Supplementary material for: Co-transfer of IncFII/IncFIB and IncFII plasmids mediated by IS26 facilitates the transmission of mcr-8.1 and tmexCD1-toprJ1
Source: Ann Clin Microbiol Antimicrob. 2024 Feb 13;23:14. doi: 10.1186/s12941-024-00676-5 (PMC10865577; doi:10.1186/s12941-024-00676-5)
Supplement: Supplementary file 1 — Additional file 1: Table S1. List of primers used in this study. [file 12941_2024_676_MOESM1_ESM.doc]

Table S1. List of primer used in this study.

| Primer | Sequence (5’-3’) | Product | Product length(bp) |
| --- | --- | --- | --- |
| Tmex-F | CTGCTGGTCATTCCGTTCCT | TmexCD1-ToprJ1 | 1196 |
| Tmex-R | ATGATCCGCTCGACGTTCTC |
| mcr8-F | TCAACAATTCTACAAAGCGTG | mcr-8.1 | 856 |
| mcr8-R | AATGCTGCGCGAATGAAG |
| Apr-F | TCGGTCAGCTTCTCAACCTT | aac(3’)-IV | 505 |
| Apr-R | ACCAACTTGCCATCCTGAAG |
