# Supplementary figures and images for: Co-transfer of IncFII/IncFIB and IncFII plasmids mediated by IS26 facilitates the transmission of mcr-8.1 and tmexCD1-toprJ1
Source: Ann Clin Microbiol Antimicrob. 2024 Feb 13;23:14. doi: 10.1186/s12941-024-00676-5 (PMC10865577; doi:10.1186/s12941-024-00676-5)

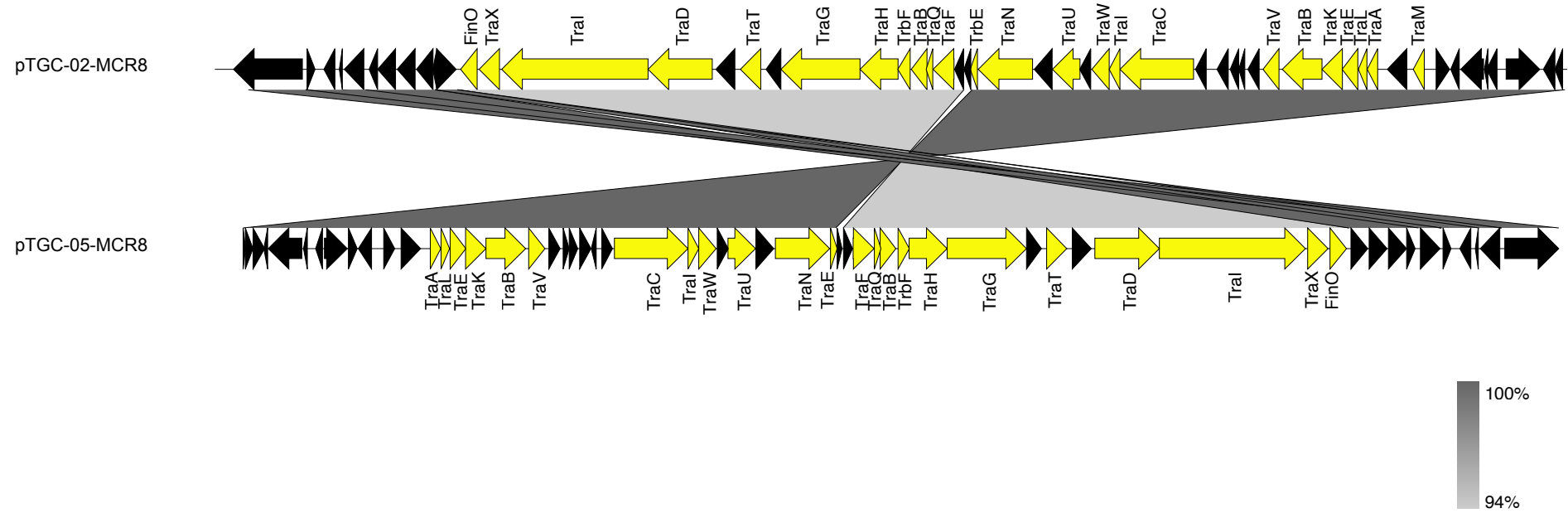

Supplement: Supplementary file 3 — Additional file 3: Figure S1. Comparative Analysis of conjugation transfer region. Alignment of the conjugation transfer region in pTGC-02-mcr8 and pTGC-05-mcr8. [file 12941_2024_676_MOESM3_ESM.pdf]
